# P-242. The Association of Steatotic Liver Disease and Cardiovascular Disease Risk in a Large Cohort of People with Human Immunodeficiency Virus

**DOI:** 10.1093/ofid/ofaf695.464

**Published:** 2026-01-11

**Authors:** Mark S Sulkowski, Alice Sternberg, Jordan Lake, Susanna Naggie, Sonya L Heath, Jennifer C Price, Laura Wilson, Holly Crandall, Samer Gawrieh, Naga Chalasani, Rohit Loomba, Richard K Sterling

**Affiliations:** Johns Hopkins University School of Medicine, Baltimore, MD; Johns Hopkins University School of Public Health, Baltimore, Maryland; University of Texas Health Science Center at Houston, Houston, TX; DCRI/ School of Medicine, Durham, NC; University of Alabama @ Birmingham, Birmingham, Alabama; University of California, San Francisco, San Francisco, CA; Johns Hopkins University School of Public Health, Baltimore, Maryland; Indiana University School of Medicine., Indianapolis, Indiana; Indiana University School of Medicine, Indianapolis, Indiana; Indiana University School of Medicine, Indianapolis, Indiana; USCD, San Diego, California; Virginia Commonwealth University, Richmond, Virginia

## Abstract

**Background:**

Steatotic liver disease (SLD), metabolic dysfunction-associated liver disease (MASLD) ± alcohol-associated liver disease (MetALD), is common in people with HIV (PWH) and associated with increased cardiovascular disease (CVD). According to SLD status, we assessed CVD risk in PWH without known CVD using validated models.

Odds ratio for 10-unit increase in risk and 95% confidence interval for 5 cardiovascular risk scores: Pairwise comparisons between steatotic liver disease (SLD) groups.

**Figure:**
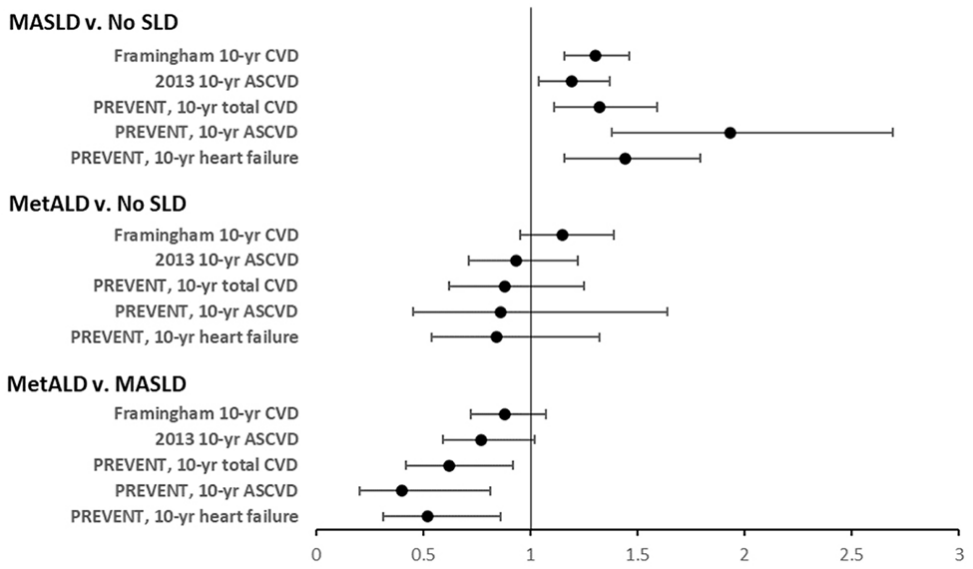

**Methods:**

Adult PWH with suppressed HIV and no heavy alcohol use were enrolled at 8 US centers. Steatosis and alcohol were assessed by elastography controlled attenuated parameter (CAP) and AUDIT, respectively. Cardiometabolic risk factors (CMRF) were BMI ≥25 kg/m^2^ or waist circumference ( >94 cm males, >80 cm females); fasting glucose ≥100 mg/dL, HbA1c ≥5.7%, type 2 diabetes, or medications; blood pressure ≥130/85 mmHg or medications; serum triglycerides ≥150 mg/dL or medications; HDL cholesterol ≤40 men /50 women mg/dL or medications. PWH were classified as: No SLD (CAP< 263 dB/m), MASLD (CAP ≥263 dB/m + ≥1 CMRF + no alcohol), or MetALD (CAP ≥263 dB/m + ≥1 CMRF + moderate alcohol ). 10-year CVD risk was assessed by three models: Framingham CVD, 2013 ASCVD, and PREVENT scores for total CVD, ASCVD, and heart failure. Those without SLD were compared to MASLD and MetALD.

**Results:**

Of 991 eligible PWH, the prevalence of MASLD was 40% and MetALD was 9%. Four CMRFs were present in 55% of those with MASLD compared to 29% without SLD. Those with MASLD had a higher mean PREVENT total CVD score than those without SLD (8.7 vs. 7.1; P=0.002) and a higher mean PREVENT ASCVD score (8.7 vs. 7.1; P=0.002) and mean PREVENT heart failure score (6.0 vs. 4.5; P=0.001). Those with MASLD also had higher mean risk than those without SLD by mean Framingham CVD score (10.4 vs. 8.8; P< 0.001) and 2013 ASCVD score (10.8 vs. 9.1; P=0.01). PWH and MetALD and those without SLD had similar CVD risk scores. Estimated high total CVD risk (≥20%) was lower with PREVENT (7%) compared to Framingham (23%) or 2013 ASCVD (12%).

**Conclusion:**

Multiple CMRFs are common in PWH and MASLD. PREVENT scores estimated higher 10-year CVD, ASCVD, and heart failure risk in those with MASLD versus those without SLD. Compared to other models, fewer PWH had high CVD risk with PREVENT. Further research is needed to validate PREVENT in PWH.

**Disclosures:**

Mark S. Sulkowski, MD, AbbVie: Advisor/Consultant|Aligos: Advisor/Consultant|Aligos: Grant/Research Support|Gilead: Advisor/Consultant|Grifols: Grant/Research Support|GSK: Advisor/Consultant|GSK: Grant/Research Support|Pfizer: Advisor/Consultant|Precision Biosciences: Advisor/Consultant|Vir: Advisor/Consultant|Vir: Grant/Research Support|Virion: Advisor/Consultant Jordan Lake, MD, ViiV: Advisor/Consultant Jennifer C. Price, MD, PhD, AbbVie: Grant/Research Support|Gilead: Grant/Research Support|VIR: Grant/Research Support Samer Gawrieh, MD, DSM: Grant/Research Support|Kowa: Advisor/Consultant|Novonordisk: Advisor/Consultant|Pfizer: Advisor/Consultant|Spruce: Advisor/Consultant|TransMedics: Advisor/Consultant|Viking: Grant/Research Support|Zydus: Grant/Research Support Richard K. Sterling, MD, MSc, Abbott: Grant/Research Support|AskBio: Advisor/Consultant|Gigagen: Grant/Research Support|Madgrigal: Advisor/Consultant|Roche: Grant/Research Support|Vertex: Advisor/Consultant|Zydus: Grant/Research Support

